# Preparation of a Molecularly Imprinted Silica Nanoparticles Embedded Microfiltration Membrane for Selective Separation of Tetrabromobisphenol A from Water

**DOI:** 10.3390/membranes13060571

**Published:** 2023-05-31

**Authors:** Xingran Zhang, Xiang Luo, Jiaqi Wei, Yuanyuan Zhang, Minmin Jiang, Qiaoyan Wei, Mei Chen, Xueye Wang, Xuehong Zhang, Junjian Zheng

**Affiliations:** 1 College of Life and Environmental Science, Guilin University of Electronic Technology, 1 Jinji Road, Guilin 541004, China; xrzhang@dhu.edu.cn (X.Z.); luoxiang123lucia@163.com (X.L.); weijiaqihere@163.com (J.W.); zhangyuanyuan0226@hotmail.com (Y.Z.); jiangminmin1015@163.com (M.J.); wqy@guet.edu.cn (Q.W.); zhangxuehong@x263.net (X.Z.); 2 School of Environmental Science and Engineering, Donghua University, 2999 North Renmin Road, Shanghai 201620, China; 3 Guangxi Key Laboratory of Automatic Detecting Technology and Instruments, Guilin University of Electronic Technology, 1 Jinji Road, Guilin 541004, China; 4 School of Environmental Science and Engineering, Nankai University, 38 Tongyan Road, Tianjin 300350, China; meichen1223@nankai.edu.cn; 5 State Key Laboratory of Pollution Control and Resource Reuse, School of Environmental Science and Engineering, Tongji University, 1239 Siping Road, Shanghai 200092, China; xiaoye@tongji.edu.cn

**Keywords:** tetrabromobisphenol A, polyvinylidene difluoride membrane, molecularly imprinted nanoparticles, molecularly imprinted membranes, selective separation

## Abstract

The ubiquitous presence of tetrabromobisphenol A (TBBPA) in aquatic environments has caused severe environmental and public health concerns; it is therefore of great significance to develop effective techniques to remove this compound from contaminated waters. Herein, a TBBPA imprinted membrane was successfully fabricated via incorporating imprinted silica nanoparticles (SiO_2_ NPs). The TBBPA imprinted layer was synthesized on the 3-(methacryloyloxy) propyltrimethoxysilane (KH-570) modified SiO_2_ NPs via surface imprinting. Eluted TBBPA molecularly imprinted nanoparticles (E-TBBPA-MINs) were incorporated onto a polyvinylidene difluoride (PVDF) microfiltration membrane via vacuum-assisted filtration. The obtained E-TBBPA-MINs embedded membrane (E-TBBPA-MIM) showed appreciable permeation selectivity toward the structurally analogous to TBBPA (i.e., 6.74, 5.24 and 6.31 of the permselectivity factors for p-tert-butylphenol (BP), bisphenol A (BPA) and 4,4′-dihydroxybiphenyl (DDBP), respectively), far superior to the non-imprinted membrane (i.e., 1.47, 1.17 and 1.56 for BP, BPA and DDBP, respectively). The permselectivity mechanism of E-TBBPA-MIM could be attributed to the specific chemical adsorption and spatial complementation of TBBPA molecules by the imprinted cavities. The resulting E-TBBPA-MIM exhibited good stability after five adsorption/desorption cycles. The findings of this study validated the feasibility of developing nanoparticles embedded molecularly imprinted membrane for efficient separation and removal of TBBPA from water.

## 1. Introduction

Endocrine-disrupting compounds (EDCs), which are ubiquitously occurring in contaminated waters, have been drawing increasing attention for their potential risks to human health, such as disrupting hormonal balance and inducing reproductive abnormality [1,2,3]. Tetrabromobisphenol A (TBBPA) (i.e., a kind of EDC)—which has been frequently detected in natural waters, both from domestic and industrial discharges—exhibits greater toxic potency due to its closer binding with the serum transport protein Transthyretin (TTR) [4]. It is therefore of significance to develop effective techniques to remove this compound from aquatic environments.

Versatile physiochemical approaches have been applied to remove/degrade TBBPA, such as advanced oxidation [5,6], electrocatalysis [7], and adsorption/adhesion [8,9]. Among them, the molecular imprinting technique shows promise mainly because of its powerful molecular recognition capability for higher removal performance of target contaminants [10]. However, the conventional molecular imprinting fabrication process, typically based on either bulk or precipitation polymerization for preparing molecularly imprinted polymers (MIPs), has limitations since the templates are easily able to leak from the polymer matrix, resulting in a poor imprinting effect.

Membrane separation technology has been employed in recent years to remove EDCs due to its efficient separation performance and environmental friendliness without secondary pollution, compared to other chemical methods [11,12]. Molecularly imprinted membranes (MIMs), fabricated by constructing the high-affinity imprinted cavities on the membranes, have become promising options for EDCs removal [13,14]. Recently, molecularly imprinted nanoparticles (MINs) have attracted much attention for the construction of MIMs because they can form sufficient, effective, and stable imprinting functional layers on the membrane surface. Various nanoparticles (NPs), such as polystyrene [15], Fe_3_O_4_ [16], and TiO_2_ [17] have been introduced to MIMs. However, previously developed MIMs for TBBPA removal were produced by directly constructing imprinted cavities on the membrane surface through complicated fabrication procedures [14,18]. In comparison to the membrane matrix, MINs are capable of providing more grafting sites for surface polymerization to form more imprinted cavities because of their large specific surface area. This prompts us to explore more simple ways for incorporating MINs onto polymeric membranes to prepare novel MIM with a more efficient removal capacity for TBBPA.

In this study, we prepared a TBBPA imprinted membrane by incorporating the imprinted silica nanoparticles (SiO_2_ NPs) onto polyvinylidene difluoride (PVDF) microfiltration membranes via vacuum-assisted filtration. SiO_2_ NPs were adopted as the supporters for imprinting TBBPA, mainly because: (i) SiO_2_ NPs are non-toxic, inexpensive, and easy to manufacture, and have a large specific surface area as well as good physicochemical stability [19]; (ii) abundant hydroxyl groups are present on the SiO_2_ NPs surface [20], which can be easily used as grafting sites to construct imprinted cavities. The TBBPA imprinted layer was synthesized on the 3-(methacryloyloxy) propyltrimethoxysilane (KH-570) modified SiO_2_ NPs by a surface imprinting approach using 4-vinylpridine (4-VP) and ethylene glycoldimethacrylate (EGDMA) as a functional monomer and cross-linker, respectively. Eluted TBBPA molecularly imprinted nanoparticles (E-TBBPA-MINs) were incorporated onto a PVDF membrane via vacuum-assisted filtration. The rebinding ability, selective separation capacity, and the mechanisms of the E-TBBPA-MINs embedded membrane (E-TBBPA-MIM) toward TBBPA were systematically investigated through the adsorption kinetics, isothermal adsorption, rebinding selectivity, and stability experiments. This study provides a promising strategy to develop molecularly imprinted membranes for the separation and removal of specific pollutants such as EDCs.

## 2. Materials and Methods

### 2.1. Chemicals

Tetrabromobisphenol A (TBBPA, 98%), ethylene glycoldimethacrylate (EGDMA, 98%), tetraethyl orthosilicate (TEOS), 3-(methacryloyloxy) propyltrimethoxysilane (KH-570, 97%), 4-vinylpridine (4-VP, 96%), 2,2′-Azobis (2-methylpropionitrile) (AIBN, 98%), bisphenol A (BPA, >99%), p-tert-Butylphenol (BP, 99%), and 4,4′-dihydroxybiphenyl (DDBP, 97%) were purchased from Sigma Aldrich (St. Louis, MO, USA). The solvents used in this study, including acetonitrile, ethanol, toluene, methanol, ammonia, and acetic acid, were obtained from China Sinopharm Chemical Reagent Co., Ltd. (Shanghai, China). In particular, TEOS was used as the raw material for preparing SiO_2_ NPs, and the KH-570 as the silane coupling agent for SiO_2_ NPs modification. TBBPA, EGDMA, 4-VP, and AIBN were adopted as the template molecule, crosslinking agent, functional monomer, and initiator for fabricating the imprinted SiO_2_ NPs, respectively. BPA, BP, and DDBP were employed as the competitive compounds of TBBPA in the selective adsorption experiments.

### 2.2. Synthesis of E-TBBPA-MINs and E-TBBPA-MIM

**Figure 1:**
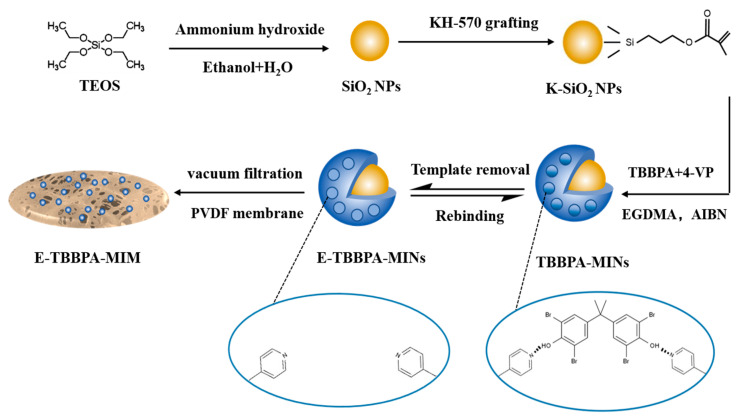
Schematic diagram of the preparation route of E-TBBPA-MINs and E-TBBPA-MIM.

SiO_2_ NPs were fabricated with the Stöber method [21]. The experimental setup for the synthesis and modification of SiO_2_ NPs is shown in Figure S1. The schematic diagram of the preparation route of E-TBBPA-MINs and E-TBBPA-MIM is exhibited in Figure 1. To prepare SiO_2_ NPs, 10 mL TEOS was added into a mixture of ammonia aqueous solution (28 wt%, 20 mL), deionized water (50 mL), and ethanol (110 mL). The mixture was stirred for 2 h. SiO_2_ NPs were then obtained after centrifugation, ethanol-washing (three times), and 12 h of drying at 60 °C. KH-570 was grafted on the surface of SiO_2_ NPs according to the reported procedures [10,22,23,24]. In brief, the SiO_2_ NPs (0.15 g) were dispersed in toluene (30 mL), followed by the slow addition of 4 mL KH-570 and stirring. Subsequently, the mixture was flushed with nitrogen for 10 min to remove oxygen and heated at 50 °C for 12 h. The obtained products (termed K-SiO_2_ NPs) were collected following centrifugation, ethanol-washing (three times), and 12 h of drying at 60 °C.

The fabrication procedure of E-TBBPA-MINs was conducted as follows: First, TBBPA (0.1 mmol) and 4-VP (0.1–0.8 mmol) were mixed in toluene (30 mL) and mechanically stirred under 30 °C for 1 h. Then, 0.15 g of K-SiO_2_ NPs were dispersed in the mixture, followed by adding EGDMA (0.8–2.4 mmol) and AIBN (20 mg). Next, the mixture reacted at 60 °C for 24 h under N_2_ protection to achieve the TBBPA molecularly imprinted nanoparticles (termed TBBPA-MINs). Subsequently, unreacted monomers absorbed on the imprinted nanoparticles were removed using ethanol-washing thrice. Finally, E-TBBPA-MINs were obtained after removing the template molecule (i.e., TBBPA) from the TBBPA-MINs with an eluent and dried at 60 °C. Note that the eluent was a mixture of acetic acid and methanol (vol./vol. = 1:9), which was commonly used for rinsing the template molecules from MIPs [25,26]. The elution process was completed until no TBBPA was detected in the eluent.

Prior to the preparation of E-TBBPA-MIM, the optimal dosage of 4-VP and EGDMA was assured by comparing the measured *Q_e_* values (i.e., equilibrium adsorption capacity in mg/g. The calculation procedure can be found in Section 2.4.1) after suspending 10 mg E-TBBPA-MINs in 50 mg/L TBBPA solution for 2 h at 25 °C. Thereafter, the E-TBBPA-MIM was fabricated using the E-TBBPA-MINs with the optimal TBBPA/4-VP/EGDMA molar ratio. In brief, 5 mg of E-TBBPA-MINs was dispersed in ethanol (50 mL), and then vacuum-filtrated onto a PVDF microfiltration membrane (pore size = 0.45 μm), followed by drying at 40 °C overnight to obtain the E-TBBPA-MIM. Note that the non-imprinted nanoparticles and membrane (without adding the TBBPA, termed NINs and NIM, respectively) were manufactured according to the same procedure.

### 2.3. Characterization of E-TBBPA-MINs and E-TBBPA-MIM

Morphologies and/or elemental compositions of E-TBBPA-MINs and E-TBBPA-MIM were determined by scanning electron microscopy equipped with an energy-dispersive spectrometer (SEM-EDS, Tescan Mira4, Brno, Czech Republic), transmission electron microscopy (TEM, JEOL F200, Tokyo, Japan), and atomic force microscopy (AFM, Bruker Dimension Icon, Bremen, Germany). The surface functional groups and chemical states of key elements of SiO_2_ NPs, E-TBBPA-MINs, and NINs were measured with a Fourier transform infrared spectrometer (FTIR, Thermo Scientific Nicolet 670, Waltham, MA, USA) and X-ray photoelectron spectroscopy (XPS, Thermo Scientific Nexsa, Waltham, MA, USA). The PerkinElmer system was utilized to perform thermo-gravimetric analysis (TGA) of SiO_2_ NPs and E-TBBPA-MINs. A Brunauer–Emmett–Teller (BET, Micromeritics ASAP 2460, Norcross, GA, USA) analysis was conducted to compare the characteristics of N_2_ adsorption/desorption isotherm, specific surface area, average pore diameter, and pore volume of E-TBBPA-MINs, and NINs. Membrane-wetting behaviors of E-TBBPA-MIM and pristine PVDF membrane were evaluated using a contact angle meter (SDC 100S, Beijing, China). Measurements of the porosity and water permeability of these two membranes were conducted according to the procedures described previously [27,28].

### 2.4. Batch Rebinding Experiments

#### 2.4.1. Adsorption Isotherms of E-TBBPA-MIM

E-TBBPA-MIM and the control membrane (NIM) were placed in a series of 30 mL TBBPA solutions with the initial concentration of TBBPA ranging from 10 to 100 mg/L (i.e., 10, 20, 40, 60, 80, and 100 mg/L). The series of mixtures were shaken for 2 h at 25 °C, and then the equilibrium concentration of TBBPA was measured by high-performance liquid chromatography (HPLC). All adsorption experiments were conducted at 25 °C, which was among the commonly used temperature range (20–35 °C) to investigate the adsorption behaviors of MIPs for organic contaminants [29,30,31]. The binding amount at equilibrium *Q_e_* was calculated by the following equation:

(1) $$Q_{e}=\frac{\left(C_{0}-C_{e}\right)V}{W}$$

where *C*_0_ (mg/L) and *C_e_* (mg/L) represent the initial and equilibrium concentration of TBBPA, respectively. *W* (mg) and *V* (mL) signify the mass of membranes and the volume of the TBBPA solution, respectively.

#### 2.4.2. Kinetic Absorption of E-TBBPA-MIM

E-TBBPA-MIM and NIM were placed in the TBBPA solution (60 mg/L) and adsorbed within a series of different times (i.e., 10, 20, 40, 60, 80, 100, 120, 150 and 180 min). After adsorption, the supernatant was analyzed by HPLC, and the absorption capacity *Q_t_* (mg/g) was calculated at each contact time (*t)* as:

(2) $$Q_{t}=\frac{\left(C_{0}-C_{t}\right)V}{W}$$

where *C_t_* (mg/L) represents the concentration of TBBPA at time *t*.

#### 2.4.3. Selective Rebinding Experiments

The selectivity of E-TBBPA-MIM was investigated in the presence of bisphenols compounds (i.e., TBBPA, BPA, BP, and DDBP) with the same initial concentration (60 mg/L). In detail, E-TBBPA-MIM and NIM were placed in the solution of TBBPA, BPA, BP, and DDBP, respectively, followed by incubation at 25 °C. The concentration of TBBPA and its structural analogues in supernatant was examined with HPLC. The adsorption capacity (*Q*, mg/g), static allocation coefficient (*K*, L/g), and imprinting factor *α* were computed according to the following equations:

(3) $$Q=\frac{\left(C_{0}-C\right)V}{W}$$

(4) $$K=\frac{Q}{C}$$

(5) $$\alpha =\frac{K_{TBBPA}}{K_{i}}\left(i=BPA,DDBP,BP\right)$$

where *C*_0_ (mg/L) and *C* (mg/L) represent the concentration of TBBPA before and after adsorption, respectively.

#### 2.4.4. Stability Tests

To investigate the regeneration and stability of E-TBBPA-MIM, the membranes were placed in TBBPA solution (60 mg/L) for adsorption (2 h, 25 °C). After each adsorption experiment, the E-TBBPA-MIM was subject to the elution process, as described in Section 2.2. The E-TBBPA-MIM was used for the subsequent five cycles (adsorption/desorption) to evaluate the regeneration capability. The turbidity of the eluents was measured by a portable turbidimeter (Hach 2100P, Loveland, CO, USA) to examine whether SiO_2_ MINs were released from the E-TBBPA-MIM.

### 2.5. Selective Permeation Experiments

A cross-flow filtration mode was conducted to evaluate the permeation performance of E-TBBPA-MIM and NIM (Figure S2). The effective filtration area of membranes was 19.6 cm^2^, and the effluent flow rate was controlled at 24 mL/min by a peristaltic pump (LeadFluid YZ 15, Baoding, China). The synthetic feeding solution contained TBBPA, BPA, BP, and DDBP (60 mg/L, 120 mL). At the predetermined time intervals, i.e., 5, 10, 20, 30, 60, 90, and 120 min, the effluent was collected to measure the concentration of TBBPA and its analogues by HPLC. The permeation flux *J* (mg/cm^2^·min), permeation coefficient *P* (L/cm·min), and permselectivity factors *β* were computed as follows:

(6) $$J=\frac{∆C_{R}V_{R}}{∆tA}$$

(7) $$P=\frac{Jd}{C_{F}-C_{R}}$$

(8) $$\beta =\frac{P_{i}}{P_{TBBPA}}\left(i=BPA,DDBP,BP\right)$$

where Δ*C_R_*/Δ*t* (mg/L·min) indicates the variation of TBBPA and its analogues concentration in effluent vs. time. *V_R_* (L), *A*(cm^2^), and *d* (cm) are the solution volume of influent, active membrane area, and thickness of the membrane, respectively. (*C_F_* − *C_R_*) (mg/L) is the concentration difference of TBBPA and its analogues between influent and effluent.

## 3. Results and Discussion

### 3.1. Optimization of E-TBBPA-MINs Composition

Appropriate dosages of functional monomers and cross-linker are crucial to the imprinting performance of MINs since they regulate the quantity and structure of recognition sites [32,33]. As shown in Figure S3A, the *Q_e_* of E-TBBPA-MINs increased with the augment of TBBPA/4-VP molar ratio in the range of 1:1 to 1:4, and further increasing this ratio resulted in the diminished *Q_e_*. This can be attributed to the fact that though a larger dosage of functional monomer can give rise to more formation of functional monomer-template complexes (i.e., the precursor of imprinted cavities), the self-association effect of excess functional monomers dooms to happen, thus decreasing the interaction between them and templates [34,35]. An adequate dosage of cross-linker, which determines the rigidity of the imprinted polymer skeleton, is a major prerequisite for producing imprinted cavities with high stability and binding capacity for templates [33]. Figure S3B shows that the optimum molar ratio of 4-VP/EGDMA was 1:5, and a higher molar ratio led to a decreased *Q_e_*. A plausible explanation is that TBBPA became difficult to elute from the imprinted polymer with an excessively high cross-linking degree, incurred by the overdose of EGDMA. Hence, the following experiments were carried out using the E-TBBPA-MINs/MIM prepared at the optimal TBBPA/4-VP/EGDMA molar ratio of 1:4:20.

### 3.2. Characterization of E-TBBPA-MINs and E-TBBPA-MIMs

**Figure 2:**
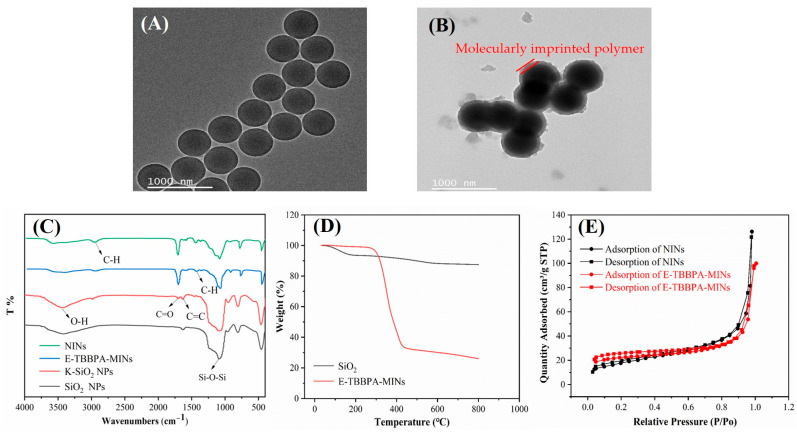
TEM images of (**A**) SiO_2_ NPs and (**B**) E-TBBPA-MINs. (**C**) FTIR spectra identifying the different functional groups of all types of NPs. (**D**) TGA curves for SiO_2_ NPs and E-TBBPA-MINs. (**E**) N_2_ adsorption-desorption isotherms of NINs and E-TBBPA-MINs.

TEM images, as shown in Figure 2A,B, present the morphologies of SiO_2_ NPs and E-TBBPA-MINs. It can be observed that SiO_2_ NPs have a regular diameter of 300 nm with good dispersibility. After TBBPA was loaded and eluted, a dense shell of 60 nm thickness representing the layer of MIPs was well coated on the surface of the SiO_2_ NPs. FTIR analysis of SiO_2_ NPs, K-SiO_2_ NPs, E-TBBPA-MINs, and NINs is illustrated in Figure 2C. The peaks around 1100 cm^−1^, representing the Si-O-Si antisymmetric stretching vibration [25], occurred for SiO_2_ NPs, K-SiO_2_ NPs, TBBPA-MINs, and E-TBBPA-MINs, suggesting that the imprinting process did not alter the characteristics of SiO_2_ NPs. In the spectrum of K-SiO_2_ NPs, the characteristic peaks of C=C and C=O were detected at 1634 cm^−1^ and 1707 cm^−1^ [36], respectively, indicating the successful grafting of KH-570 on the surface of SiO_2_ NPs. The presence of EGDMA can be confirmed by the appearance of a stretching vibration adsorption peak at 1438 cm^−1^ [33]. The EDS analysis results of the elemental composition of all NPs are shown in Table 1. With the introduction of TBBPA, the amount of nitrogen and bromine increased significantly to 0.6% and 0.2%, respectively, while after the elution of TBBPA, the bromine content decreased to 0%. In line with this, as can be observed from the comparison of XPS C1s spectra of TBBPA-MINs and E-TBBPA-MINs, only the C-Br peak at the binding energy of 287.2 eV disappeared after the elution (Figure S4A,B). These results corroborated that the elution process was capable of completely removing TBBPA without destroying the polymeric network of MIPs.

The influence of the imprinting process on the thermal stability of SiO_2_ NPs was investigated by TGA from 20 to 800 °C (Figure 2D). It can be observed that E-TBBPA-MINs exhibited three distinct weight loss stages. Figure 2D shows that ~74% of weight loss occurred in the second stage between 280 and 450 °C, likely associated with the decomposition of MIPs on the surface of SiO_2_ NPs [37,38]. In the first and third stages, the weight loss process of E-TBBPA-MINs might be ascribed to the disappearance of residual water and carbonization of polymer chains on the surface of SiO_2_ NPs, respectively [39]. Figure 2E shows the N_2_ adsorption-desorption isotherms of E-TBBPA-MINs and NINs. It can be inferred that both NPs have similar mesoporous structures, given their physisorption isotherms with the Type IV and H3 hysteresis loop features. Furthermore, it can be seen from Table S1 that the specific surface area, average pore size, and pore volume of E-TBBPA-MINs and NINs were quite similar, implying that compared to NINs, the pore structure might not be an important factor influencing the adsorption ability of E-TBBPA-MINs.

**Figure 3:**
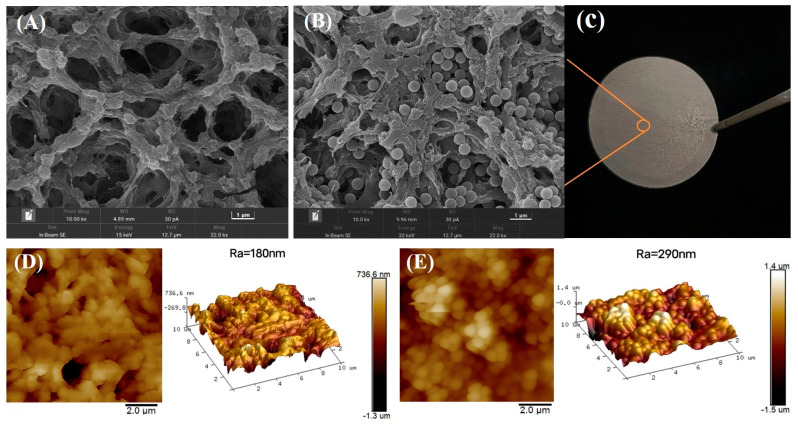
SEM images of (**A**) the pristine PVDF membrane and (**B**) E-TBBPA-MINs embedded mem brane (E-TBBPA-MIM). (**C**) photograph of E-TBBPA-MIM. AFM images and relative average roughness (*R*_a_) of (**D**) the pristine PVDF membrane and (**E**) E-TBBPA-MIM.

Figure 3A–C presents the surface morphologies of E-TBBPA-MINs embedded membrane and the pristine PVDF membrane (i.e., E-TBBPA-MIM). It can be seen that for the E-TBBPA-MIM, a large quantity of spherical NPs was closely attached onto the pore walls of the membrane matrix, suggesting the successful loading of E-TBBPA-MINs. Furthermore, after the immobilization of E-TBBPA-MINs, compared to the pristine membrane, the average surface roughness (*R*_a_) of E-TBBPA-MIM significantly increased by ~61%, as shown in Figure 3D,E. Table 2 shows the membrane intrinsic properties of E-TBBPA-MIM and the pristine PVDF membrane. The introduction of E-TBBPA-MINs had a remarkably positive impact on the hydrophilicity of E-TBBPA-MIM, given the smaller contact angle of E-TBBPA-MIM than pristine PVDF membrane (14.3 ± 1.6° vs. 97.5 ± 1.7°). This can be ascribed to the coverage of the hydrophilic SiO_2_ NP_S_ on the membrane surface. However, significant changes in the membrane permeability and porosity were also observed, suggesting that the coverage of NPs on the membrane surface might block up the pores. The equilibrium adsorption capacity (*Q_e_*) of E-TBBPA-MIM reached 19.2 ± 0.3 mg/g, significantly greater than that (0.5 ± 0.2 mg/g) of the pristine PVDF membrane.

### 3.3. Adsorption Performance of E-TBBPA-MIM

**Figure 4:**
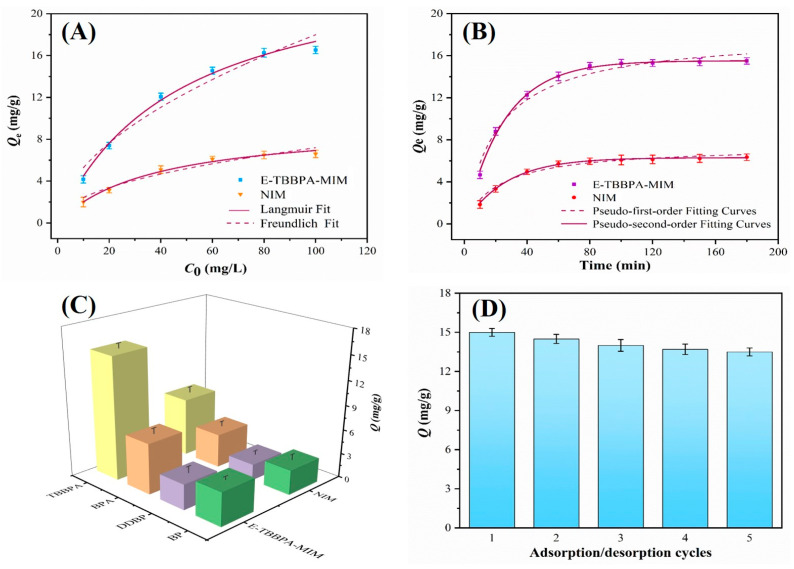
Adsorption isotherms (**A**) and kinetics (**B**) isotherms for E-TBBPA-MIM and NIM. (**C**) Selective adsorption results of E-TBBPA-MIM and NIM. (**D**) Stability of the E-TBBPA-MIM after exposure to varying adsorption/desorption cycles.

The adsorption capacities of E-TBBPA-MIM and NINs embedded PVDF membrane (NIM) toward TBBPA (10–100 mg/L) were investigated by HPLC. It can be observed from Figure 4A that in the case of all tested TBBPA concentrations, the *Q_e_* values of E-TBBPA-MIM were much higher than that of NIM, which could be due to the existence of high-affinity recognition sites on E-TBBPA-MINs for TBBPA molecules [40]. In contrast, the randomly arranged functional groups on the NINs could not specifically bind the template molecules [41], thus the NIM did not possess the imprinting effect. Moreover, the *Q_e_* of E-TBBPA-MIM for TBBPA increased with increasing TBBPA concentration from 10 to 80 mg/L, attributed to the enhanced diffusion of TBBPA toward the recognition sites.

To further evaluate the adsorption mechanisms, the Langmuir (9) and Freundlich (10) model equations [42,43] were used to assess the equilibrium adsorption data of E-TBBPA-MIM and NIM:

(9) $$Q_{e}=\frac{K_{L}Q_{m}C_{e}}{1+K_{L}C_{e}}$$

(10) $$Q_{e}=K_{F}C_{e}^{1/n}$$

where *Q_e_* (mg/g) and *Q_m_* (mg/g) indicate the equilibrium and maximum rebinding capacity of TBBPA, respectively. *C_e_* (mg/L) is the equilibrium concentration of TBBPA. *K*_L_ (L/mg), *K_F_* [(mg/g)·(L/mg)^1/n^] are the Langmuir and Freundlich binding coefficients, respectively. *n* is the Freundlich binding constant. As shown in Figure 4A and Table S2, it can be seen that the linear regression values of E-TBBPA-MIM and NIM fitted better with Langmuir model (*R*^2^ = 0.9926 and 0.9882 for E-TBBPA-MIM and NIM, respectively) than those of Freundlich model (*R*^2^ = 0.9594 and 0.9478 for E-TBBPA-MIM and NIM, respectively). This can be ascribed to the homogeneous distribution of E-TBBPA-MINs on the E-TBBPA-MIM, which was consistent with the observation from SEM image in Figure 3B, because Freundlich isotherm is more suitable to study the adsorption behaviors occurring in heterogeneous system [44]. Moreover, the better accuracy of Langmuir model validated that the adsorption of TBBPA on the E-TBBPA-MIM was a monolayer adsorption process.

The adsorption kinetics analysis of TBBPA on the E-TBBPA-MIM and NIM were conducted to elucidate the rate-controlling and rebinding mechanism of TBBPA molecules. Thus, the plots of Figure 4B were fitted by the pseudo-first-order (Equation (11)) and pseudo-second-order models (Equation (12)):

(11) $$Q_{t}=Q_{e}-Q_{e}e^{-K_{1}t}$$

(12) $$Q_{t}=\frac{K_{2}Q_{e}^{2}t}{1+K_{2}Q_{t}t}$$

where *Q_e_* and *Q_t_* are the amounts of TBBPA adhered in the case of rebinding equilibrium and different time *t* (mg/g), respectively. *K*_1_ and *K*_2_ represent the equilibrium rate constants of the pseudo-first-order and pseudo-second-order kinetic models (g/mg min), respectively. It can be observed from Figure 4B and Table S3 that the rebinding capacity of E-TBBPA-MIM increased with the increase in time at the beginning. Subsequently, the curve arrived at a plateau over 60 min, mainly due to the fact that the recognition sites of E-TBBPA-MIM were almost saturated after a quick adsorption process (within one hour). In contrast, the NIM exhibited a similar curve but was inferior regarding rebinding capacity. The fitting obtained was better with the pseudo-second-order model (*R*^2^ = 0.9938) rather than pseudo-first-order model (*R*^2^ = 0.9673), implying that the adsorption behavior of TBBPA on E-TBBPA-MIM was controlled by chemical adsorption [45].

To examine the selectivity of E-TBBPA-MIM, the adsorption capabilities of E-TBBPA-MIM and NIM for TBBPA and competitive molecules (BP, BPA and DDBP) were evaluated. It can be seen from Figure 4C that E-TBBPA-MIM showed higher adsorption capacity of TBBPA than those of BP, BPA and DDBP. The imprinting factors (*α*) of BP, BPA and DDBP were 4.52, 2.88 and 6.07, respectively, suggesting that E-TBBPA-MIM had no selectivity toward non-template molecules. In contrast, there was no significant difference shown between the adsorption capabilities of E-TBBPA-MIM and NIM for competitive molecules. Figure 4D clearly indicates that the rebinding capacity of E-TBBPA-MIM still reached around 90% that of the initial after 5 adsorption/desorption cycles, demonstrating its remarkable adsorption stability. Likewise, previous studies reported that the rebinding capacities of the developed MIMs merely decreased by 7–12% after 4–6 times reuse [37,41]. In addition, during the stability tests, no turbidity could be detected in the eluents, indicating the tight coverage of SiO_2_ MINs on the E-TBBPA-MIM.

### 3.4. Permselectivity Performance and Mechanism of E-TBBPA-MIM

**Figure 5:**
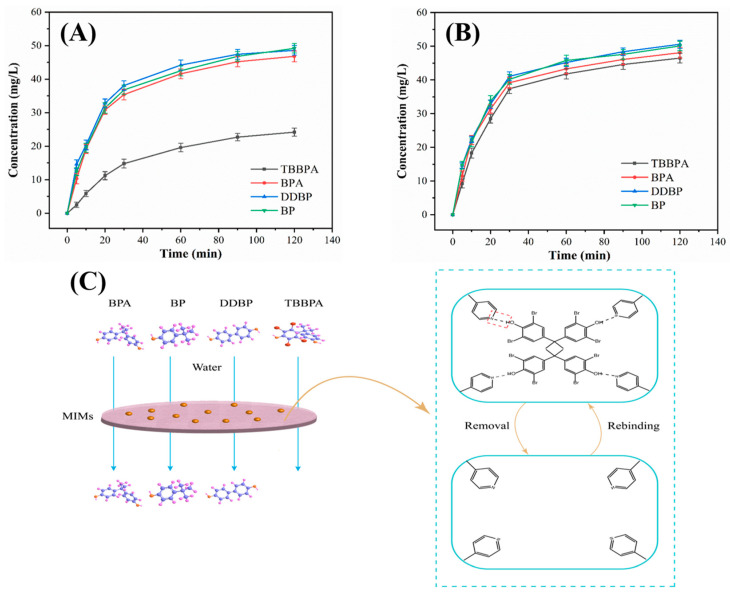
Permselectivity of TBBPA, BP, BPA, and DDBP through (**A**) E-TBBPA-MIM and (**B**) NIM. (**C**) Schematic diagram of the permselectivity mechanism of E-TBBPA-MIM toward TBBPA.

Permselectivity behaviors toward the template molecule, i.e., TPPBA, were elucidated to better understand the specific adsorption separation properties of E-TBBPA-MIM. Time-dependent permselectivity behaviors of E-TBBPA-MIM and NIM for TBBPA were investigated using BP, BPA, and DDBP as the competitive molecules (Figure 5A,B). As shown in Figure 5A,B, at any time interval, E-TBBPA-MIM and NIM resulted in the similar effluent concentrations of BP, BPA, and DDBP, indicating that the imprinted cavities on the E-TBBPA-MIM showed no specific recognition capacity toward the structural analogues of TBBPA, since either the presence or absence of imprinted cavities did not influence the permeation behaviors of these compounds. This is unsurprising because molecules, which cannot be specifically recognized by the MIMs, are known to penetrate the membranes by diffusion/convection [46]. It can be also observed that the E-TBBPA-MIM exhibited significantly greater separation performance for TBBPA than other molecules, but there was no obvious difference between the separation performance of NIM for all the types of molecules. This may be due to the fact that there existed no specific sites on the NIM available for TBBPA adsorption, thus TBBPA penetrated the membranes with other molecules in the same manner [37]. As depicted in Table S4, the permselectivity factors (*β*) of E-TBBPA-MIM toward BP, BPA, and DDBP were 6.74, 5.24, and 6.31, much greater than those of NIM (i.e., 1.47, 1.17 and 1.56 for BP, BPA, and DDBP, respectively), indicating the successful construction of specific recognition sites, which contributed to the excellent separation performance of E-TBBPA-MIM for TBBPA.

The selective separation behaviors of MIMs are often associated with two opposite mechanisms, i.e., facilitated and retarded permeation mechanisms [46,47]. Given the strong retention capacity of E-TBBPA-MIM for TBBPA, as corroborated by the results of above permselectivity experiments, the retarded permeation mechanism played the dominant role in the separation process of E-TBBPA-MIM for TBBPA. The proposed permselectivity mechanism of E-TBBPA-MIM is shown in Figure 5C. There are two reasons accounting for the selective separation of E-TBBPA-MIM for TBBPA. On one hand, the hydrogen bond interaction between the N atoms in the benzene ring of MIPs and hydroxyl groups of TBBPA resulted in the chemical adsorption of TBBPA by the imprinted cavities. For another, the chemical structure of TBBPA was perfectly matched to the shape, size, and spatial arrangement of imprinted cavities. The rebinding capacity of E-TBBPA-MIM could be recovered after the TBBPA removal by the eluent. The BP, BPA, and DDBP, which are nonmatched to the spatial structure of imprinted cavities, could not enter into the cavities to produce a hydrogen bond interaction with MIPs. Therefore, the E-TBBPA-MIM showed poor adsorption capacity for these structural analogues of TBBPA.

## 4. Conclusions

In this study, a novel TBBPA imprinted SiO_2_ NPs embedded PVDF microfiltration membrane (E-TBBPA-MIM) with enhanced permselectivity and rebinding capacity were successfully developed. The adsorption capacity of E-TBBPA-MIM for TBBPA was markedly greater than the pristine PVDF membrane and NIM. Adsorption data of TBBPA on the E-TBBPA-MIM fitted well with the Langmuir and pseudo-second-order models, suggesting that the adsorption process was a monolayer chemical adsorption process. Especially, the permselectivity factors of E-TBBPA-MIM for structural analogues (i.e., BP, BPA and DDBP) of TBBPA reached 5.24–6.71, significantly greater than those (1.17–1.56) of NIM. The rebinding capacity of E-TBBPA-MIM could still reach around 90% that of the initial after 5 adsorption/desorption cycles. The excellent adsorption performance, permselectivity and stability indicated the developed E-TBBPA-MIM as a promising option for efficient separation and removal of TBBPA from water.

## Figures and Tables

**Table 1:** Elemental composition of SiO_2_ NPs, K-SiO_2_ NPs, TBBPA-MINs, E-TBBPA-MINs, and NINs.

Type of NPs	Elemental Composition (at.%)				
	C	N	O	Si	Br
SiO_2_ NPs	0.6	-	65.7	33.6	-
K-SiO_2_ NPs	29.4	-	51.5	19.0	-
TBBPA-MINs	53.8	0.6	32.5	12.9	0.2
E-TBBPA-MINs	50.4	0.5	36.2	12.9	-
NINs	52.7	0.6	33.7	13.0	-

**Table 2:** Intrinsic properties of E-TBBPA-MIM and the pristine PVDF membrane.

	Water Permeability ^a^ (L/m^2^·h)	Contact Angle (^o^)	Porosity (%)	*Q_e_* (mg/g)
E-TBBPA-MIM	722.0 ± 14.0	14.3 ± 1.6	49.3 ± 1.0	19.2 ± 0.3
PVDF membrane	1256.0 ± 15.0	97.5 ± 1.7	72.4 ± 1.1	0.5 ± 0.2

^a^ Water permeability was tested at 30 kPa.

## Data Availability

The data presented in this study are available on request from the corresponding author.

## Supplementary Materials

The following supporting information can be downloaded at: https://www.mdpi.com/article/10.3390/membranes13060571/s1, Figure S1: Picture of experimental setup for synthesis and modification of SiO_2_ NPs; Figure S2: Picture of experimental setup for cross-flow filtration tests; Figure S3: Effects of (A) TBBPA/4-VP and (B) 4-VP/EGDMA molar ratios on *Q_e_* of MINs; Figure S4: XPS C1s spectra of (A) TBBPA-MINs and (B) E-TBBPA-MINs; Table S1: Specific surface area, average pore size and total pore volume of E-TBBPA-MINs and NINs; Table S2: Isotherm adsorption constants of E-TBBPA-MIM and NIM for TBBPA; Table S3: Kinetics constants for adsorption of TBBPA onto E-TBBPA-MIM and NIM; Table S4: Selective permeation parameters of E-TBBPA-MIM and NIM for TBBPA and its structural analogues.

## Abbreviations

TBBPA	Tetrabromobisphenol A
SiO_2_ NPs	Silica nanoparticles
KH-570	3-(methacryloyloxy) propyltrimethoxysilane
E-TBBPA-MINs	Eluted TBBPA molecularly imprinted nanoparticles
PVDF	Polyvinylidene difluoride
E-TBBPA-MIM	Eluted TBBPA molecularly imprinted membrane
BP	P-tert-butylphenol
BPA	Bisphenol A
DDBP	4,4′-dihydroxybiphenyl
EDCs	Endocrine disrupting compounds
TTR	Transthyretin
MIMs	Molecularly imprinted membranes
MINs	Molecularly imprinted nanoparticles
MIPs	Molecularly imprinted polymers
4-VP	4-vinylpridine
EGDMA	Ethylene glycol dimethacrylate
TEOS	Tetraethyl orthosilicate
AIBN	2,2′-azobis (2-methylpropionitrile)
K-SiO_2_ NPs	KH-570 modified silica nanoparticles
SEM-EDS	Energy-dispersive spectrometer
TEM	Transmission electron microscopy
AFM	Atomic force microscopy
FTIR	Fourier transform infrared spectrometer
XPS	X-ray photoelectron spectroscopy
TGA	Thermo-gravimetric analysis
BET	Brunauer-Emmett-Teller
SDC	Contact angle meter
NINS	Non-imprinted nanoparticles
NIMS	Non-imprinted membranes
HPLC	High performance liquid chromatography
*Q_e_*	Equilibrium adsorption capacity
*Q_t_*	Absorption capacity at different contact time
*Q*	Adsorption capacity
*K*	Static allocation coefficient
*α*	Imprinting factor
*J*	Permeation flux
*P*	Permeation coefficient
*β*	Permselectivity factors
*R*_a_	Roughness
