# Supplementary material for: Preparation of a Molecularly Imprinted Silica Nanoparticles Embedded Microfiltration Membrane for Selective Separation of Tetrabromobisphenol A from Water
Source: Membranes (Basel). 2023 May 31;13(6):571. doi: 10.3390/membranes13060571 (PMC10305625; doi:10.3390/membranes13060571)
Supplement: Supplementary file 1 [file membranes-13-00571-s001.zip › membranes-2411603-supplementary.pdf]

Supplementary Material

Preparation of a Molecularly Imprinted Silica Nanoparticles Embedded Microfiltration Membrane for Selective Separation of Tetrabromobisphenol A from Water

Xingran Zhang [1,2,3,†], Xiang Luo [1,†], Jiaqi Wei [1], Yuanyuan Zhang [1,3], Minmin Jiang [1,3], Qiaoyan Wei [1,3], Mei Chen [4], Xueye Wang [5], Xuehong Zhang [1,3] and Junjian Zheng [1,3,*]

[1] College of Life and Environmental Science, Guilin University of Electronic Technology, 1 Jinji Road, Guilin 541004, China; xrzhang@dhu.edu.cn (X.Z.); luoxiang123lucia@163.com (X.L.); weijiaqihere@163.com (J.W.); zhangyuanyuan0226@hotmail.com (Y.Z.); jiangminmin1015@163.com (M.J.); wqy@guet.edu.cn (Q.W.); zhangxuehong@x263.net (X.Z.)

[2] School of Environmental Science and Engineering, Donghua University, 2999 North Renmin Road, Shanghai 201620, China

[3] Guangxi Key Laboratory of Automatic Detecting Technology and Instruments, Guilin University of Electronic Technology, 1 Jinji Road, Guilin 541004, China

[4] School of Environmental Science and Engineering, Nankai University, 38 Tongyan Road, Tianjin 300350, China; meichen1223@nankai.edu.cn

[5] State Key Laboratory of Pollution Control and Resource Reuse, School of Environmental Science and Engineering, Tongji University, 1239 Siping Road, Shanghai 200092, China; xiaoye@tongji.edu.cn

* Correspondence: zhengjunjianglut@163.com or zhengjunjian@guet.edu.cn; Tel.: +86-773-2305206

† These authors contributed equally to this work.

Legends:

Figure S1. Picture of experimental setup for synthesis and modification of SiO_2 NPs.

Figure S2. Picture of experimental setup for cross-flow filtration tests.

Figure S3. Effects of (A) TBBPA/4-VP and (B) 4-VP/EGDMA molar ratios on Q_e of MINs.

Figure S4. XPS C1s spectra of (A) TBBPA-MINs and (B) E-TBBPA-MINs.

Table S1. Specific surface area, average pore size and total pore volume of E-TBBPA-MINs and NINs.

Table S2. Isotherm adsorption constants of E-TBBPA-MIM and NIM for TBBPA.

Table S3. Kinetics constants for adsorption of TBBPA onto E-TBBPA-MIM and NIM.

Table S4. Selective permeation parameters of E-TBBPA-MIM and NIM for TBBPA and its structural analogues.

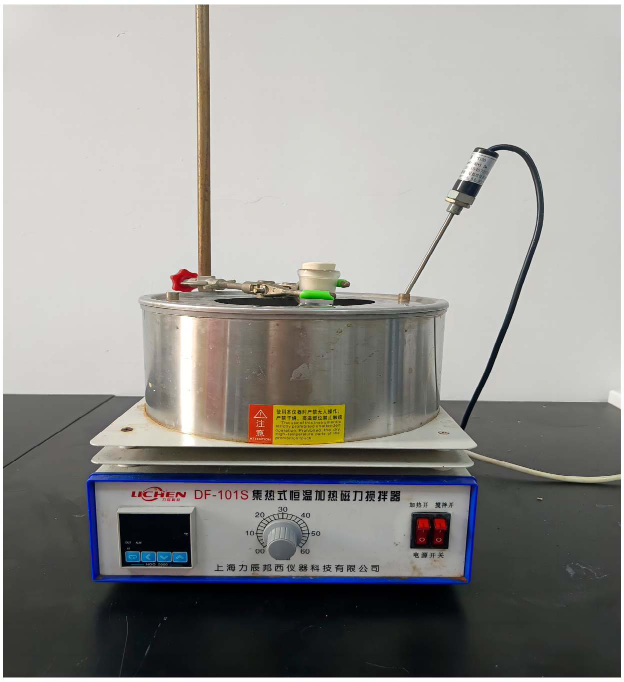

Figure S1. Picture of experimental setup for synthesis and modification of SiO_2 NPs.

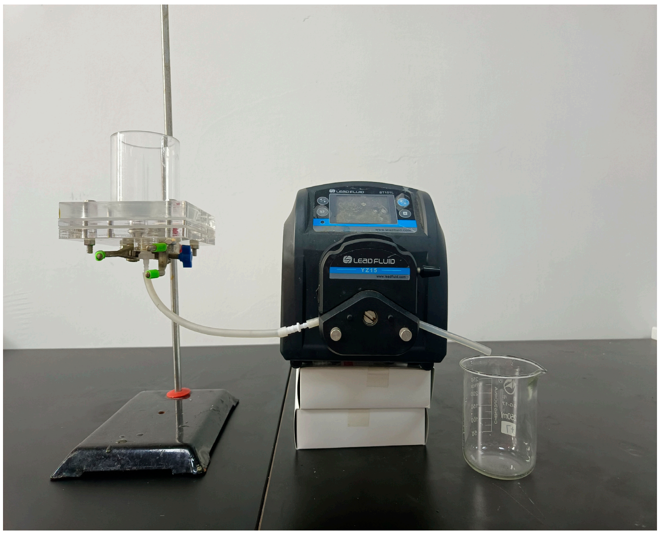

Figure S2. Picture of experimental setup for cross-flow filtration tests.

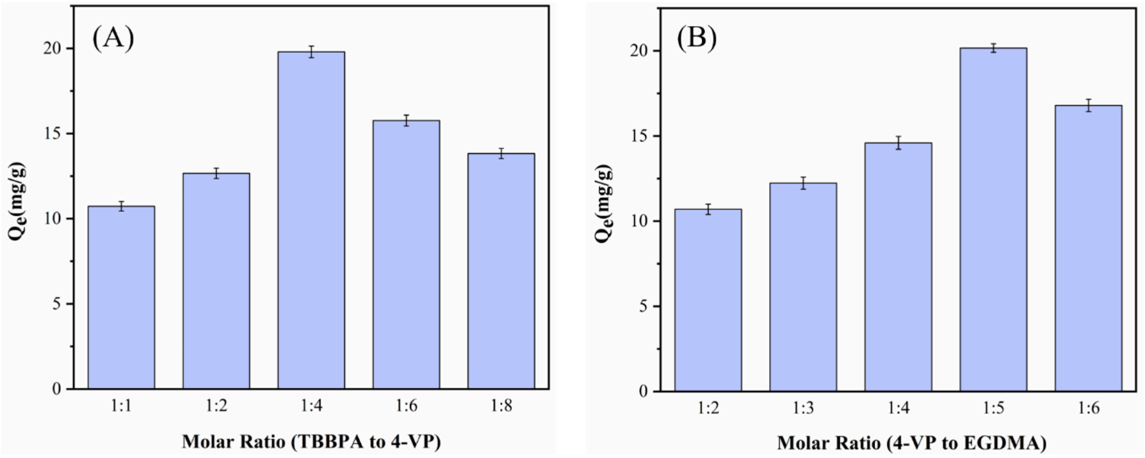

Figure S3. Effects of (A) TBBPA/4-VP and (B) 4-VP/EGDMA molar ratios on *Q*e of MINs.

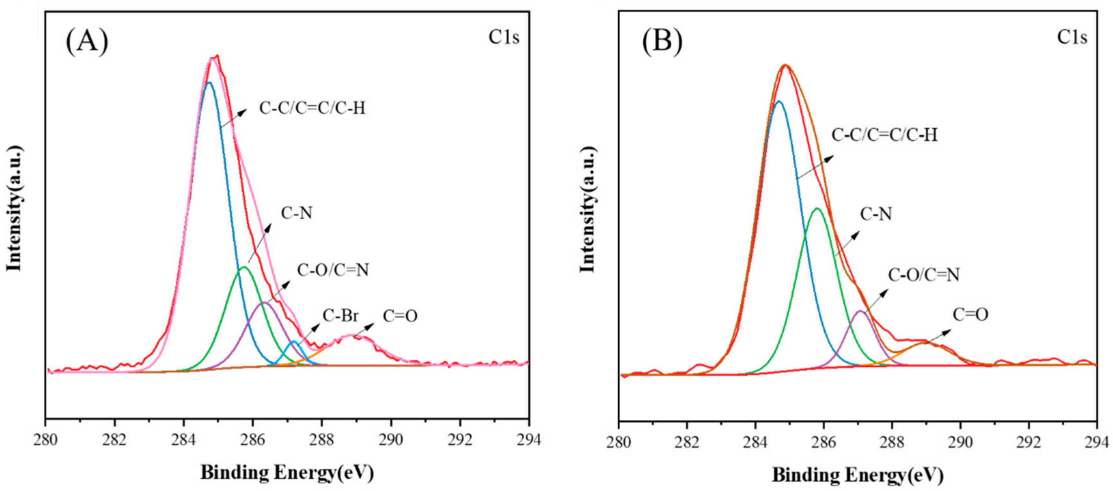

Figure S4. XPS C1s spectra of (A) TBBPA-MINs and (B) E-TBBPA-MINs.

Table S1. Specific surface area, average pore size and total pore volume of E-TBBPA-MINs and NINs.

Sample	Surface Area(m^2/g)	Pore Volume(cm^3/g)	Pore Size(nm)
E-TBBPA-MINs	66.1	0.148	8.9
NINs	52.3	0.135	7.9

Table S2. Isotherm adsorption constants of E-TBBPA-MIM and NIM for TBBPA.

Membranes	$Q_{e,exp}$[a]	Langmuir model			Freundlich model		
		$Q_{e,cal}$[b]	K_L	R^2	1/n	K_F	R^2
E-TBBPA-MIM	16.53	22.57	0.0387	0.9926	0.5298	1.8151	0.9594
NIM	6.60	8.83	0.0360	0.9882	0.5080	0.7435	0.9478

[a] $Q_{e,exp}$ (mg/g) is the experimental value of Q_e (mg/g).

[b] $Q_{e,cal}$ (mg/g) is the calculated value of Q_e (mg/g) by the Langmuir adsorption model.

Table S3. Kinetics constants for adsorption of TBBPA onto E-TBBPA-MIM and NIM.

Membrane	$Q_{e,exp}$[a]	Pseudo-first-order model			Pseudo-second-order model		
		$Q_{e,cal}$[b]	K_1	R^2	$Q_{e,cal}$[b]	K_2	R^2
E-TBBPA-MIM	15.50	12.16	0.0350	0.9673	17.57	0.0030	0.9938
NIM	6.35	4.21	0.0258	0.9558	7.21	0.0066	0.9946

[a] $Q_{e,exp}$ (mg/g) is the experimental value of Q_e (mg/g).

[b] $Q_{e,cal}$ (mg/g) is the calculated value of Q_e (mg/g).

Table S4. Selective permeation parameters of E-TBBPA-MIM and NIM for TBBPA and its structural analogues.

Membrane	Molecule	Jx10^3 (mg/min·cm^2)	Px10^6 (L/min·cm)	β
E-TBBPA-MIM	TBBPA	4.93	1.51	-
	BPA	9.53	7.94	5.24
	DDBP	9.90	9.55	6.31
	BP	10.02	10.21	6.74
NIM	TBBPA	9.47	7.72	-
	BPA	9.80	9.06	1.17
	DDBP	10.31	12.06	1.56
	BP	10.21	11.34	1.47
